# SDGs in corporate responsibility reporting: a longitudinal investigation of institutional determinants and financial performance

**DOI:** 10.1007/s10997-023-09671-y

**Published:** 2023-03-09

**Authors:** Ambra Galeazzo, Toloue Miandar, Michela Carraro

**Affiliations:** 1 Dipartimento di Scienze Economiche e Aziendali “Marco Fanno”, Università degli Studi di Padova, Via del Santo 33, Padova, Italy; 2 Dipartimento di Scienze Aziendali - DISA, Alma Mater Studiorum Università di Bologna, Bologna, Italy

**Keywords:** Sustainability, SDGs, Corporate responsibility, Corporate responsibility reporting, Financial performance

## Abstract

Companies play a central role in the achievement of Sustainable Development Goals (SDGs); as such, they face institutional pressures to increase their engagement with SDGs. However, given the complexity of SDGs, it is unclear whether these pressures lead firms to adopt engagement approaches that address a few goals or the whole set of 17, and if that choice has any subsequent effect on financial performance. To shed light on these issues, this research draws on the neo-institutional theory to investigate whether two institutional determinants—industry type and country of origin—affect SDG engagement and whether such engagement improves financial performance. Based on a content analysis and a regression analysis on high-reputation companies (the 100 most sustainable firms in the world) over the period 2017–2020, we find that the institutional pressures associated with industry type and country-of-origin positively impact any engagement approach to SDGs. However, we establish that companies’ financial performance only generally improves when engaging with either the whole set of SDGs or a specific subset of the most frequently cited. This study provides important theoretical and practical contributions that illuminate firms’ institutional and financial rationales for adopting SDGs.

## Introduction

Compared to their peers, sustainable companies are more likely to gain greater legitimacy, reduce their environmental and social risks, improve their image and reputation, and gain access to more and better resources (Barnett & Salomon, 2012; Cordeiro et al., 2018, 2021; Hussain et al., 2018). There is great value in adopting a proactive stance toward sustainability. Recently, companies are facing a new, ambitious challenge to foster their sustainable proactivity: specifically, they are asked to drive the success of the sustainable development goals (SDGs) (UN News, 2015). The SDGs represent an important shift in developing and implementing sustainable initiatives. With their 17 goals and 169 targets proposed by the United Nations (UN) General Assembly, the SDGs aim to jointly improve an interconnected set of sustainable development themes. Prior references to sustainable development—such as the Millennium Development Goals (MDGs)—involved governmental, regional, and national stakeholders. SDGs, instead, require that also companies engage as change agents by applying “their creativity and innovation to solving sustainable development challenges” (UN General Assembly, 2015, p. 29). As a result, companies increasingly face institutional pressures to assume a central role in SDGs.

These institutional pressures stem from stakeholders who want to address companies’ adoption of certain decisions and actions. According to the neo-institutional theory (Chizema & Buck, 2006; DiMaggio & Powell, 1983), companies tend to tackle sustainable issues, such as SDGs, to gain legitimacy and obtain the social license to operate (Demuijnck & Fasterling, 2016). In particular, companies tend to respond to similar institutional pressures by mimicking other companies’ decisions and actions (in line with stakeholders’ expectations). In other words, institutional pressures within similar environments, such as country of origin and industry (DiMaggio & Powell, 1983; van Zanten & van Tulder, 2018; Zhu & Sarkis, 2007), tend to encourage similar responses. Although this initiative has attracted sizable attention from different institutional environments (Haffar & Searcy, 2018; PwC, 2019), little is known about the real contribution of institutional pressures on the level of SDG engagement. Previous studies mostly drew on early adopters and cross-sectional analyses to investigate said contribution, ultimately producing insufficient and conflicting results (Elalfy et al., 2021; Silva, 2021; van der Waal & Thijssens, 2020).

The present research addresses this gap, in line with the neo-institutional view, by exploring two types of institutional environments. Specifically, we distinguish between developed countries vs. developing countries, assuming that stakeholders in the former are more concerned about SDGs. Furthermore, we consider the industrial environment by distinguishing between high-polluting industries vs. less-polluting industries, assuming that stakeholders in the former may be more sensitive to SDGs. We choose these two institutional environments to explore whether their different pressures foster different SDG engagement strategies. Indeed, given the internal complexity of the SDGs, companies have great discretion in tailoring their strategic approach in response to their respective institutional pressures. As a result, they may allocate resources to a broad range of goals or a focused combination of them (e.g., environmental-related goals, social-related goals). For example, stakeholders in developing countries are more likely to compel companies to invest in a focused combination of SDGs (e.g., social-related SDGs) rather than the overall set of SDGs. In contrast, stakeholders in high-polluting industries may be more concerned with environmental-related SDGs than the total set. Past studies have overlooked this issue: most of them have investigated a small number of individual goals rather than the complete set (Magliacani, 2022; Mio et al., 2020; Sullivan et al., 2018). Our study overcomes this problem by acknowledging the various strategic approaches to SDG engagement, which may be similar among companies that share an institutional environment and thus are more likely to adopt a similar strategic approach as a response.

It is important to note that companies’ engagement in SDGs may be symbolic or substantive. According to the literature, a symbolic response implies a superficial effort to fulfill institutional requests, whereas a substantive response involves a costly and effortful change in the design and management of strategies and processes (Adams & Frost, 2008; Maas et al., 2016). Past studies have highlighted this issue in the case of SDGs, questioning whether companies’ initiatives contribute to a concrete change in their strategies and operations (Bebbington & Unerman, 2018; Elalfy et al., 2021). To explore whether companies’ engagement in SDGs creates more incentive for symbolic or substantive change, we study the effect of SDGs on financial performance. In doing so, we assume that stakeholders can distinguish between symbolic and substantive responses (Schons & Steinmeier, 2016), and thus will only reward companies that substantively transform their businesses to fully address SDGs.

Overall, our research provides insights into the following questions: (a) how do institutional pressures affect companies’ engagement in the overall set of SDGs vs. specific subsets of SDGs; and (b) to what extent do institutional pressures toward SDGs’ engagement lead, in turn, to credible, substantive initiatives that positively affect financial performance? Our study addresses these questions by adopting both qualitative and quantitative analyses. Compared to previous studies focusing on samples selected from the Fortune Global 250 (e.g., Ehnert et al., 2016; Perego & Kolk, 2012) or national databases (e.g., Uyar 2017), we focus on organizations with a high reputation for sustainability performance. The rationale for this choice is that SDGs are a relatively recent creation; many companies are still debating how to include them in their strategy (PwC, 2019). The risk is to have a scarce understanding of the development of SDGs adoption as organizations do not report them yet. As high-reputation companies are those that constantly meet stakeholders’ expectations (Petkova et al., 2014), they are more likely to promptly modify their sustainability strategy and integrate new regulations (such as SDGs) as they arise. High-reputation companies may also serve as a benchmark for the evolution of SDGs in other organizations.

We, therefore, study the sustainability reports of the “global 100 most sustainable corporations in the world” (Corporate Knights, 2020) over the period 2017–2020. We then perform a qualitative analysis using the NVivo software to investigate the extent to which companies disclose their SDG engagement. On this point, we identify four strategies: (1) level of engagement in SDGs as a whole, (2) level of engagement in environmental-related SDGs, (3) level of engagement in social-related SDGs, and (4) level of engagement in the most frequent combination of SDGs pursued by organizations. Finally, we perform a quantitative analysis using longitudinal regression models to investigate the effect of the selected institutional pressures (i.e., developed vs. developing countries; high-polluting vs. low-polluting industry) on the four strategies, and by extension, their effects on financial performance. Our findings show that companies in developing countries and high-polluting industries are more engaged with SDGs compared to companies in developed countries and low-polluting industries, without any difference among the strategic approaches to SDGs. On the contrary, the strategic approaches to SDGs become important to explain whether SDGs engagement is considered substantive and, therefore, yields positive financial implications. Specifically, we find that only an engagement in the overall set of SDGs or the focused set of SDGs 8 (decent work and economic growth), 13 (climate action), and 12 (responsible consumption and production) has a positive and significant effect on financial performance.

## Literature review and hypotheses development

### SDGs: definition and relevance in the management literature

The SDGs are built on long-term deliberations between countries and the UN. The origins of SDGs date back to the Earth Summit in Brazil in 1992, where Agenda 21 was adopted. Eight years later, the Millennium Summit at the UN headquarters in New York solidified a vision to reduce extreme poverty by 2015 through the implementation of MDGs. In 2002, the World Summit on Sustainable Development in Africa included more emphasis on multilateral partnerships. At the Rio + 20 conference in 2012, the outcome document “The Future We Want” sought to develop a set of SDGs based on the MDGs that were adopted by the Member States. A key difference between MDGs and SDGs is that the latter applies to both developing and developed countries. In 2013, the Open Working Group was created to develop a proposal on the SDGs. This eventually culminated in the 2030 Agenda for Sustainable Development in September 2015, wherein the UN General Assembly (UN Department of Economic and Social Affairs-Sustainable Development) established 17 SDGs and 169 targets. Learning from the mistakes of the MDGs, Agenda 2030 tracks the results annually and utilizes intermediate milestones (Sachs, 2012).

SDGs are an urgent call for action addressed to all actors in society, but they are especially important to for-profit companies, which play a strategic role in countries’ economic, social, and environmental progress (Berrone et al., 2019; Mio et al., 2020). As such, it is important to deepen our understanding of what motivates companies to achieve the SDGs and whether this engagement is perceived as a substantive response to sustainability-related challenges, thus fostering financial performance. Meyer and Rowan (1977) emphasized that companies’ decisions may be dictated as much or more by their institutional environment than by a business rationale. Therefore, we adopt an institutional lens in the hopes of generating interesting insights into the antecedents of companies’ SDG engagement.

### Institutional pressures on SDG engagement

The neo-institutional theory is widely recognized as an important theoretical framework for investigating companies’ sustainable initiatives. Indeed, firms face increasing pressure to address environmental, ethical, and social issues in response to evolving government action and societal norms (e.g., Crane & Matten, 2016; Rivera, 2010; Swanson, 2018; van Zanten & van Tulder, 2018; Wijen et al., 2012). According to neo-institutional theory (DiMaggio & Powell, 1983), firms of the same institutional context gradually converge on a set of strategic and organizational dimensions due to three mechanisms. First, isomorphism may derive from pressures exerted either by the dependency on other organizations or by the host society’s cultural expectations (i.e., coercive isomorphism). Second, organizations may adopt mimetic behaviors to cope with technological and environmental uncertainties and ambiguities (i.e., mimetic isomorphism). Third, management professionalization tends to create homogeneity in terms of education and career paths, as managers are often rewarded for helping the organization reach competitors’ level of performance (i.e., normative isomorphism).

Given its ability to explain companies’ convergence toward a set of strategic and organizational decisions, the neo-institutional theory has been widely utilized in the context of sustainability. For instance, in the analysis of annual reports from 45 Canadian firms, Bansal (2005) found that media pressures (as one institutional force) are important in the early years of corporate sustainable development, but their relevance is likely to decrease over time. Meanwhile, Marano and Kostova (2016) showed that multinational enterprises base their adoption of CSR decisions on the multiplicity of institutional forces. Furthermore, in a survey of 72 Brazilian firms, Galleli et al. (2021) demonstrated that in periods of crisis, such as the COVID-19 pandemic, coercive pressures from governmental regulations strongly influence companies’ SDG strategy. These applications of neo-institutional theory signal its utility for explaining why and how companies engage in sustainable development.

Companies respond to institutional requisites and stakeholder concerns by disclosing sustainability-related information (e.g., the level of engagement in SDGs) in their annual reports (Rasche et al., 2013; Young & Marais, 2012). Because companies can choose which goals to address, recent studies have highlighted some factors that orient firms’ investments (Stevens & Kanie, 2016). For example, Van Zanten and van Tulder (2018) drew on a cross-sectional survey of 86 global companies to explore their commitment to SDG targets. They found that global companies headquartered in Europe engaged more with SDG targets than those based in North America, preferring to engage in SDG targets that “avoid harm” rather than on proactive initiatives that “do good”. Furthermore, Tsalis et al. (2020) uncovered that Greek companies disclose information on issues such as the use of renewable energy, investment in infrastructures and innovation, and the reduction of greenhouse gas emissions; however, they are less transparent when reporting their policies for protecting human rights and eliminating corruption incidents. These findings suggest that Greek companies have a preference for environmental-related goals. In a similar vein, Gunawan et al. (2020) analyzed SDG reporting attitudes in Indonesian companies. The authors found that Indonesian companies are more likely to disclose information about good health and well-being, quality education, and sustainable cities and communities, but less likely to provide information about gender equality, clean water and sanitization, and zero hunger. In other words, Indonesian companies seem to show a greater commitment to social-related goals. Considered together, these findings suggest that companies headquartered in the same country converge on their engagement strategies while diverging from companies headquartered in other countries.

However, the past literature has not identified whether the country of origin exerts institutional pressures that compel companies to engage with SDGs. In the present study, we address Mio et al. (2020) and Bashir and Qureshi (2022) call for distinguishing between developing vs. developed countries to explain how country-of-origin encourages companies to disclose a broader range of SDGs in CSR reporting. Such a distinction is important because institutional pressures in developing vs. developed countries are quite dissimilar due to institutional voids. Specifically, institutional voids arise in developing countries due to weak governmental, legal, and monitoring mechanisms, which limit the pressure on companies (Cordeiro et al., 2018, 2021). Those countries may also abstain from applying pressure due to the fear that sustainability-related requirements could have negative economic and employment effects (Earnhart et al., 2014). On the contrary, in developed countries where governmental and social pressures are strong, stakeholders have the power to force companies to prove their commitment toward SDGs. Furthermore, extant studies demonstrate that multinational enterprises headquartered in developed countries tend to engage in SDGs in developing countries as a strategy to address institutional pressures in their country of origin and improve their position in society (Ghauri, 2022; van Tulder et al., 2021). Overall, these arguments suggest that companies in developed countries are more likely to engage in SDGs (i.e., overall SDGs; environmental-related SDGs; social-related SDGs; a focused combination of SDGs) relative to companies headquartered in developing countries. Formally:

#### H1

Companies in developed countries are more likely to engage in SDGs than companies in developing countries.

We also hypothesize that industrial sectors may explain the extent to which companies converge in their SDG engagement. Because organizations are increasingly required to minimize their environmental footprint and adopt green strategies aimed at pollution abatement (Gunawan et al., 2020; Li et al., 2018; Liute & de Giacomo, 2022), we expect that institutional pressures are higher in industries that record higher levels of pollution. Companies in these industries are probably more prone to demonstrating their efforts to minimize their unsustainable behaviors. For instance, in a study on 420 European B Corps, Alonso-Martínez et al. (2020) provided evidence that the greater the pollution level of a company, the higher the attention it pays to society and the environment. Similarly, we suggest that high-polluting industries tend to disclose a broader range of SDGs in an attempt to compensate for their negative impact on the environment. Therefore, we hypothesize that:

#### H2

Companies in high-polluting industries are more likely to engage in SDGs than companies in low-polluting industries.

### The effects of SDGs engagement on financial performance

The literature on sustainability has widely discussed whether such initiatives are substantive or symbolic (Schons & Steinmeier, 2016; Wang et al., 2018). Indeed, scholars have argued that, as substantive initiatives are costly to implement, companies are more likely to commit their resources and competencies for their success, contributing to the transformation of the business as usual. The distinction between symbolic and substantive thus illuminates why certain sustainability-related initiatives are sometimes unable to contribute to greater legitimacy, reduced environmental and social risks, improved image and reputation, and access to more and better resources (Barnett & Salomon, 2012), the culmination of which would eventually lead to a positive influence on financial performance (Cordeiro et al., 2021; Hussain et al., 2018).

A symbolic purpose means that companies declare an investment in SDGs without triggering a change in their current practices—that is, they maintain “business as usual.” On the contrary, having a substantive purpose implies that companies undergo the “values-based business” transformation when investing in SDGs (Sebhatu & Enquist, 2022). Some scholars cast doubt on the substantive initiatives underlying SDGs (Elalfy et al., 2020; Silva 2021). On the one hand, Silva (2021) studied FTSE 100 reports on companies’ sustainability performance and found that they predominantly engage with SDGs in a symbolic way. Indeed, they focus on either existing or future sustainable plans to describe their links to SDGs, while failing to identify the specific, quantitative targets associated with each sustainable action and decision. On the other hand, Elalfy et al. (2020) analyzed 24,000 tweets about SDGs from Standard and Poor 500 companies, finding that companies take SDGs seriously because they incorporate these goals into their core business. Overall, there is no agreement on either the symbolic or substantive nature of SDGs.

We hypothesize that firms’ engagement in SDGs may be perceived as either substantive or symbolic depending on the chosen strategic approach. Because engagement is costly—requiring a full alignment of the firm’s resources, competencies, and strategies with sustainability—we argue that only a strong engagement in SDGs (i.e., the whole set of SDGs) can support a sustainability-related transformation. Thus, we hypothesize that the more companies engage in SDGs (i.e., overall SDGs are preferred to environmental-related SDGs, social-related SDGs, and a focused combination of SDGs), the higher their financial performance. Formally:

#### H3

A greater engagement in SDGs is positively associated with financial performance.

## Methodology

### Data collection and sample

This study analyzes the sustainability reports of the “global 100 most sustainable corporations in the world” based on the Corporate Knights index. Corporate Knights is a Certified B Corp that publishes one of the world’s most widespread business magazines on sustainability, alongside rankings of firms’ corporate sustainability performance. Other researchers have employed the Corporate Knights index. For example, Ramos et al. (2022) examined the SDG coverage of international firms in six industries listed in the Corporate Knights index, including banking, insurance, petroleum refineries, real estate investment and services, and investment services. Similarly, Henry et al. (2019) evaluated 22 global energy companies in the index across a period of 11 years, finding that the presence of a chief sustainability officer does not boost triple-bottom-line performance.

Since 2005, Corporate Knights has selected publicly listed companies with gross revenues above $1 billion based on data from Bloomberg and sustainability reports (approximately 4000 firms). This list is then narrowed to 100 companies using a screening procedure involving product types (companies producing products that harm the natural environment or human lives, such as tobacco and weapons, are automatically excluded), sustainable behaviors (companies that lobby to block sustainability-related policies are excluded), financial strength, payouts due to sanctions, fines or settlements on sustainability issues, and the extent to which companies disclose information on a set of indicators such as pollution releases, waste productivity, gender equality, innovation capacity, and other social, environmental, and financial aspects. The shortlisted companies receive a score on a set of up to 21 performance indicators, some of which differ for each industry type and are measured against peers of the same industry (for more details, visit https://www.corporateknights.com/reports/2020-global-100/). The global 100 ranking is released every year at the World Economic Forum in Davos. The 2020 global 100 ranking comprises corporations from 25 countries and 41 sectors (both service and manufacturing) around the world, thus providing a fair representation of mid-to-large international companies (see Tables 1 and 2).

**Table 1:** Sample composition by headquarters locations

Location	Frequency	Percentage	Cumulative (%)
USA	17	17	17
Canada	12	12	29
France	8	8	37
Denmark	6	6	43
Japan	6	6	49
Finland	5	5	54
Germany	5	5	59
UK	5	5	64
Italy	4	4	68
Netherlands	3	3	71
Brazil	3	3	74
Sweden	3	3	77
China	3	3	80
Others	20	20	100

**Table 2:** Sample composition by headquarters locations

Industry	Frequency	Percentage	Cumulative (%)
36 Electronic & other electric equipment	10	10	10
60 Bank	10	10	20
28 Pharmaceutical	9	9	29
50 Auto	9	9	38
49 Electric, gas, & sanitary services	8	8	46
35 Industrial machinery & equipment	7	7	53
73 Business services	6	6	59
38 Healthcare	4	4	63
51 Chemicals	4	4	67
65 Real Estate	4	4	71
87 Engineering & management services	4	4	75
20 Food	3	3	78
67 Holding & other investment offices	3	3	81
Others	19	19	100

We visited the websites of the global 100 most sustainable corporations to collect sustainability or integrated annual reports released during the period 2017–2020. We chose these 4 years because the Agenda 2030 containing the SDGs was decided in September 2015 and came into force on January 1, 2016. As annual reports generally disclose information on initiatives occurring in the previous year, the sustainability reports released in 2017 are likely to encompass initiatives started in 2016. In approximately 25% of the total number of cases, sustainability or CSR reports were not available and thus we analyzed the sustainability or CSR section in the annual report. It is worth noting that the number of companies publishing dedicated sustainability reports has increased over the years.

## Measures

### Disclosure of the SDGs

The research is grounded on a mixed qualitative and quantitative approach. On the qualitative side, we applied the content analysis methodology to firms’ SDG disclosures (i.e., the number of SDGs disclosed by each firm of the sample in their reports). Content analysis codifies narrative data into various categories based on specified criteria. Such narrative data refers to words, symbols, ideas, themes, meanings, pictures, or messages that can be communicated in documents (Gunawan et al., 2020). Codifying that data is integral to creating quantitative scales for use in empirical models. Content analysis has been widely applied in previous research analyzing voluntary disclosure (Michelon & Parbonetti, 2012). We conducted the following procedure. First, we used the NVivo software to code the sustainability reports or integrated annual reports of the 100 sampled companies from 2017 to 2020. We employed the sentence as the recording unit and we checked whether it contained: “SDG” followed by the number (e.g., either including the space “SDG 1”, “SDG 2” or without the space as in “SDG1” and “SDG2”); “sustainable development goal” followed by the number (e.g., Sustainable Development Goal 1); or the exact wording of each of the 17 SDGs (e.g., for SDG 1: “No poverty”). The software matched every sentence with each one of the 17 SDGs and coded the sentence as 0 if no information about a specific SDG was included, 1 otherwise. If other sentences in the report repeated information on the same SDG, we counted this information only once. Second, after codification, we created a database with dummy variables for each SDG disclosed in each report by each firm in each year.

We created different measures of SDG disclosure. TotSDGs is the sum of the SDGs disclosed by each firm in a specific year. EnvSDGs is the sum of the SDGs disclosed by each firm in a specific year regarding environmental goals. We classified the following SDGs as environmental goals: SDG6 (Clean water and sanitation), SDG7 (Affordable and clean energy), SDG13 (Climate action), SDG14 (Life below water), SDG15 (Life on land), and SDG12 (Responsible consumption and production). SocSDGs is the sum of the SDGs disclosed by each firm in a specific year regarding social goals. We classified the following SDGs as social goals: SDG1 (No poverty), SDG2 (Zero hunger), SDG3 (Good health and well-being), SDG4 (Quality education), SDG5 (Gender equality), SDG8 (Decent work and economic growth), SDG10 (Reduced inequalities), SDG11 (Sustainable cities and communities), and SDG16 (Peace, justice and strong institutions). We excluded SDG9 (Industry, innovation, and infrastructure) and SDG17 (Partnerships for the goals) from both groups because they are not related to environmental or social initiatives. Finally, frequentSDGs is the sum of SDG8, SDG12, and SDG13, which are the most addressed SDGs based on the qualitative analysis reported in the next section. This variable was created to understand whether a specific group of SDGs can generate superior financial performance.

### Institutional variables

We collected data on the institutional variables from the reports. Developed country is a dummy variable that takes a score of 1 if the firm is headquartered in a developed country, 0 otherwise. We distinguished between developed vs. developing countries based on the World Economic Situation and Prospects (2019), developed by the United Nations. Firms with headquarters in Australia, the European Union, Japan, North America, Norway, Switzerland, and the United Kingdom were classified as located in developed countries. We also included the provinces of Hong Kong, Singapore, and Taiwan in this category because of their high income. Firms with headquarters in Brazil, China, Mexico, South Africa, and South Korea were classified as located in developing countries.

High-polluting industries is a dummy variable that takes the value of 1 if the firm belongs to the most polluting industries, 0 otherwise. Following Kanashiro (2020), high-polluting industries are those that tend to exceed polluting thresholds defined by the Environmental Protection Agency (EPA)—specifically, under the EPA’s Toxics Release Inventory (TRI) program. Following the approach of previous authors (Berrone & Gomez-Mejia, 2009; King & Lenox, 2002), we measured environmental performance as total toxic emissions, as reported to the TRI. Using their primary two-digit SIC codes, firms in the electronic products, electric utilities, machinery, chemicals, food, petroleum, transportation equipment, merchant wholesalers, and miscellaneous manufacturing were classified as belonging to high-polluting industries (Kanashiro, 2020). The other industries (banks, pharmaceuticals, auto, business services, healthcare, real estate, engineering, and management services, and investment management) were classified as low-pollution industries. In our sample, there were 54 firms in the former category and 46 firms in the latter.

### Financial variable

Financial performance was measured using the return on assets (ROA) retrieved from Thomson Reuters Datastream. ROA is an accounting-based financial metric that captures the firm’s past short-term performance. It is calculated as profit before interest and tax, divided by total assets. It is one of the most-used performance indicators in the management literature (Murphy et al., 1996). We led the variable by 1 year to capture the causal effect of SDG disclosure at year t on the financial performance of year t + 1.

### Control variables

As bigger firms may face more institutional pressures and stakeholder scrutiny, we first controlled for firm size, measured as the natural logarithm of number of employees. We also controlled for the report type. As firms in the dataset disclose sustainability information either in dedicated sustainability reports or an integrated annual report, we created a dummy variable taking the value of 1 if the firm discloses sustainability information in integrated annual reports, 0 if the firm uses sustainability reports. Compared to sustainability reports that provide an overview of the firm’s initiatives in the environmental, social, and economic areas, integrated annual reports follow the guidelines of the International Integrated Reporting Council (IIRC) to communicate how their strategy, governance, performance, and prospects create value over the short-, medium-, and long-term. Such a difference in communication style may affect SDGs’ disclosure.

### Data analysis

We performed both qualitative and quantitative data analyses. The former drew on content analysis to create the variables related to the SDGs’ engagement (i.e., overall SDGs, environmental-related SDGs, social-related SDGs, and a focused combination of SDGs). The quantitative analysis tested our hypotheses using panel data regression in an unbalanced panel dataset. We used unbalanced data because many firms in the dataset lacked information on SDGs and financial performance in 2020. We used the Hausman-Wu test to decide between a fixed effect and a random effect. The results indicated that a random-effect model was appropriate [χ2 = 2.44; | P = 0.118]. We also used the Wooldridge test (Drukker, 2003; Wooldridge, 2002) to verify whether there was a problem with first-order autocorrelation in our panel data [F = 3.95| P = 0.167]. Finally, we tested for panel-level heteroskedasticity using the likelihood-ratio test after estimation, which affirmed a lack of heteroskedastic problems [F = 4.44| p = 0.000].

## Results

### Descriptive statistics

Based on the content analysis performed using NVivo, we can illustrate the different SDGs that every company promoted between 2017 and 2019, immediately following the launch of Agenda 2030. As only a few firms released their reports in 2020, we did not consider them in this qualitative analysis. The quantitative results related to SDGs, which were based on the generated dummy variables, offer an overall view of the number of addressed SDGs in every report. On the other hand, through the qualitative coding of reports also the focus of companies on different SDGs based on high, medium, and low levels of attention was captured. Specifically, if a company had taken one or a set of SDGs into account as the main focus of their contribution, more details were provided for those SDGs and more reference codes were assigned to those SDGs in the report.

**Fig. 1:**
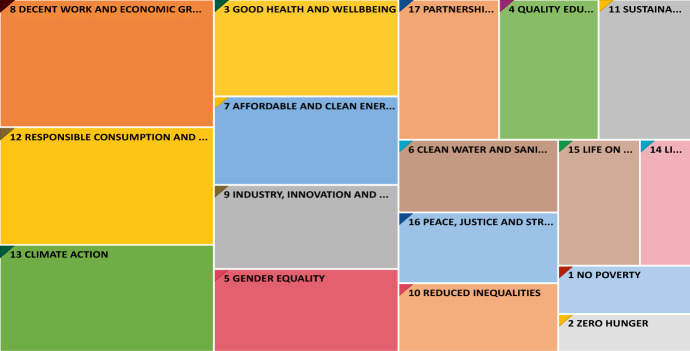
Most addressed 17 SDGs over 2017–2019

Figure 1 offers a general illustration of how the world’s 100 most sustainable companies have addressed SDGs over the past four years. One can see that companies paid the most attention to SDG8 (Decent work and economic growth), SDG12 (Responsible consumption and production), and SDG13 (Climate action), but the least attention to SDG1 (No poverty), SDG2 (Zero hunger), and SDG14 (Life below water).

**Fig. 2:**
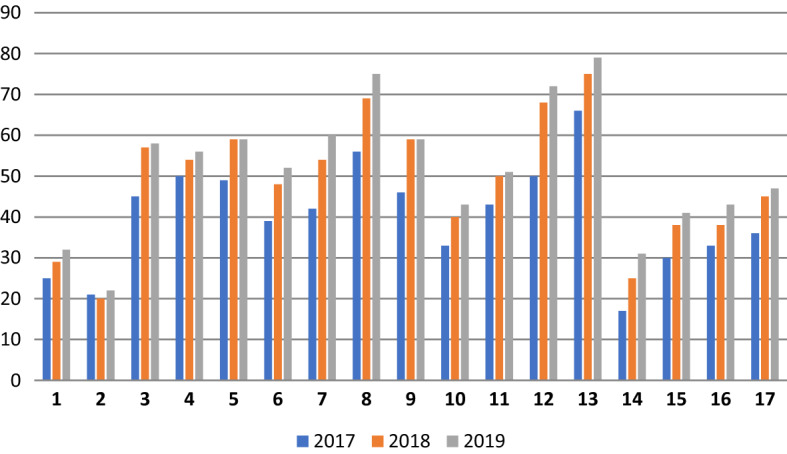
Total number of SDGs disclosed in the overall sample in all years (2017–2019)

Figure 2 shows how each of the 17 SDGs was addressed in 2017, 2018, and 2019, as well as how the SDGs’ inclusion in the reports changed over those 3 years. The overall number of addressed SDGs gradually increased from 2017 to 2019, with most of the companies starting with the SDGs most relevant to their business. For example, energy companies began by focusing on SDG7 (affordable and clean energy). Over time, these companies gradually expanded their efforts to a greater number of SDGs in their sustainability reports.

**Fig. 3:**
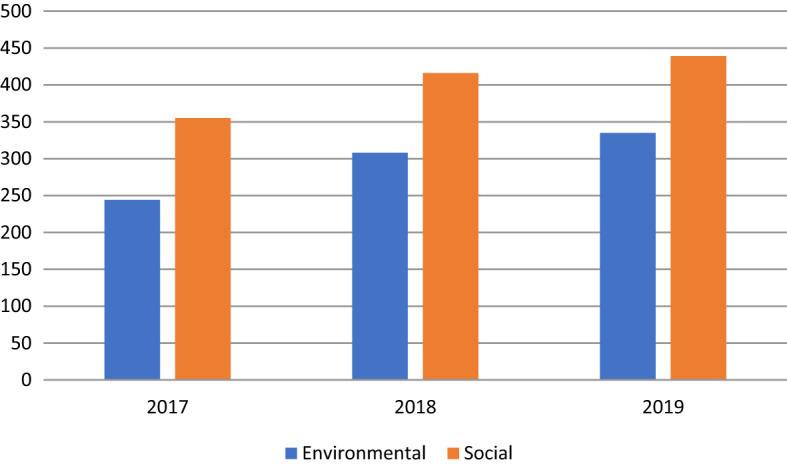
Two categories of social and environmental SDGs from 2017 to 2019

Figure 3 represents how the two categories of envSDGs and socSDGs evolved from 2017 to 2019. In both categories, the number of addressed SDGs increased across the years, but the social category saw an overall higher number of disclosures. It appears that socSDGs (vs. envSDGs) are less (vs. more) measurable and therefore easier (vs. more challenging) to report on.

**Fig. 4:**
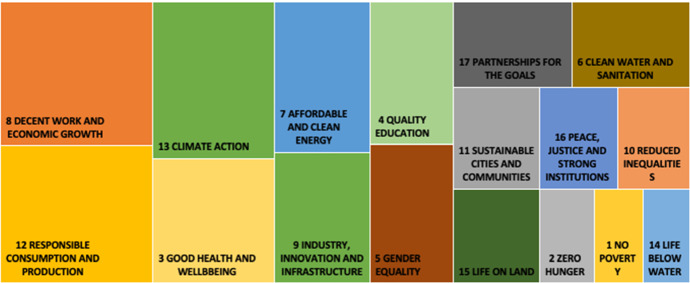
Most addressed SDGs over 2017–2019 for the most polluting industries

**Fig. 5:**
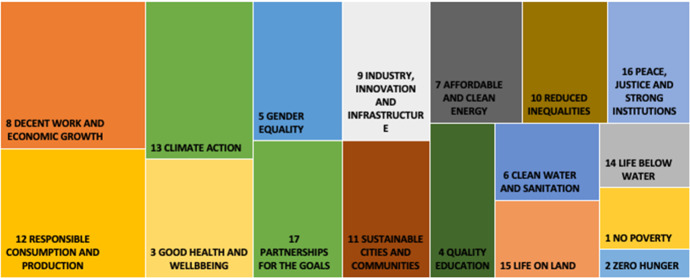
Most addressed SDGs over 2017–2019 for less polluting industries

Figures 4 and 5 show the most disclosed SDGs in high- and low-polluting industries, respectively, across the observation period. In both industry groups, the four most addressed SDGs were SDG3, SDG8, SDG12, and SDG13, which aligns with the overall sample (Fig. 1). After this point, the groups diverge in interesting ways. Specifically, in high-polluting industries, the fifth- and sixth-most addressed SDGs are SDG7 (Affordable and clean energy) and SDG9 (Industry innovation and infrastructure); whereas in low-polluting industries, the comparable goals are SDG5 (Gender equality) and SDG17 (Partnership for the goals).

**Fig. 6:**
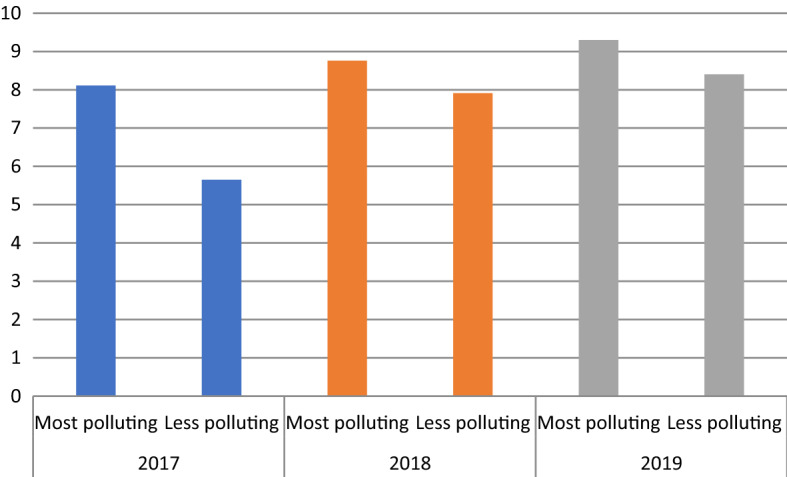
Average number of SDGs disclosed in each industry category over the period 2017–2019

**Fig. 7:**
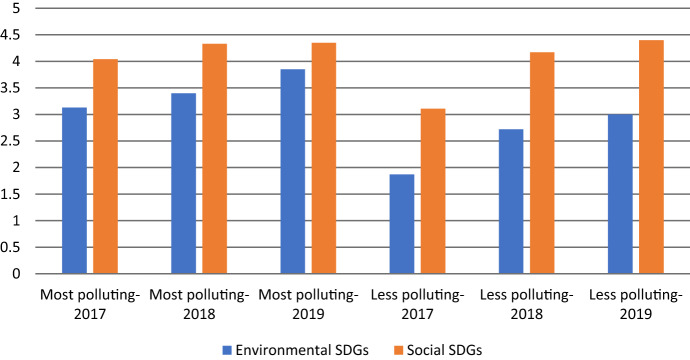
Average number of envSDGs and socSDGs in each industry category over the period 2017–2019

In Fig. 6, we present the average number of disclosed SDGs in each industry category over the years 2017–2019. Firms in high-polluting industries are more engaged in the disclosure of SDGs compared to their low-polluting counterparts. Figure 7 shows the average number of disclosed envSDGs and socSDGs in each industry category. Even though in both industry types socSDGs were more disclosed than envSDGs, low-polluting industries demonstrated a more visible increase in addressing both types of SDGs over the years.

### Empirical analysis

Table 3 reports the results of the panel data regression analysis regarding the effects of institutional pressures (proxied by the variables *developed countries* and *high-polluting industries*) on engagement with the whole set of disclosed SDGs (TotSDGs). Model 1 is the baseline that solely includes the control variables. Model 2 adds developed countries and high-polluting industries to the baseline model. The estimated coefficient of the developed countries variable is negative and significant (b = − 3.695, p < 0.01), in contrast with H1. This result indicates that firms in developing countries disclose more SDGs in their reports compared to firms in developed countries. However, the estimated coefficient of the high-polluting industries is positive and significant (b = 1.729, p < 0.01), thus supporting H2. This result highlights that firms in high-polluting industries tend to disclose SDGs in their reports more proactively than firms in low-polluting industries.

**Table 3:** Regression results for the antecedents of SDGs disclosure

	TotSDGs^(a)^	TotSDGs^(a)^
	Model 1	Model 2
Developed countries		− 3.695***
		(1.198)
High polluting industries		1.729***
		(0.628)
Size	0.416**	0.414**
	(0.204)	(0.202)
Report type	− 0.638	− 0.376
	(0.729)	(0.710)
Constant	4.316**	6.727***
	(2.044)	(2.220)
Observations	294	294
R-squared	0.016	0.072
Adj. R-squared	0.009	0.060

*p < 0.10; **p < 0.05; ***p < 0.01

^(a)^The results are similar in terms of significance and sign when the other measures of SDGs disclosure (EnvSDGs, socSDGs, frequentSDGs) are used

Table 4 reports the results of the panel data regression analysis regarding the effects of different measures of SDG engagement on financial performance. Model 1 includes the control variables. Model 2 adds the totSDGs variable to Model 1. Model 3 adds the socSDGs and envSDGs variables to Model 1. Model 4 adds the frequentSDGs variable to Model 1. The results in Model 2 indicate that an extended engagement with SDGs has a positive and significant impact on financial performance (b = 0.004, p < 0.1). However, the results in Model 3 indicate that the strategy of focusing on the disclosure of social SDGs or environmental SDGs does not pay: neither type had a positive and significant impact on financial performance. However, the results in Model 4 show that a focused disclosure of SDG8 (Decent work and economic growth), SDG12 (Responsible consumption and production), and SDG13 (Climate action) has a positive and significant effect on financial performance. Overall, these findings partially support H3 and suggest that only some strategic approaches to SDG engagement are profitable.

**Table 4:** Regression results for the financial impact of SDGs implementation

	ROA			
	Model 1	Model 2	Model 3	Model 4
TotSDGs		0.004*		
		(0.003)		
SocSDGs			0.012	
			(0.009)	
EnvSDGs			− 0.004	
			(0.013)	
FrequentSDGs				0.022**
				(0.010)
Size	0.023**	0.020**	0.019**	0.0209**
	(0.00946)	(0.00905)	(0.00867)	(0.009)
Report type	0.0390	0.0403	0.0423	0.040
	(0.0304)	(0.0305)	(0.0304)	(0.031)
Developed countries	0.00595	0.0236	0.0240	0.022
	(0.0350)	(0.0355)	(0.0359)	(0.034)
High polluting industries	0.0201	0.0115	0.0139	0.010
	(0.0281)	(0.0269)	(0.0269)	(0.027)
Constant	− 0.0936	− 0.116	− 0.107	− 0.130
	(0.106)	(0.110)	(0.107)	(0.110)
Observations	169	169	169	169
R-squared	0.032	0.046	0.050	0.048
Adjusted R-squared	0.00810	0.0169	0.0151	0.0187

*p < 0.10; **p < 0.05; ***p < 0.01

## Discussion

SDGs were developed to help companies become more proactive in addressing the world’s compounding sustainable challenges. Nevertheless, the past literature has failed to explain how effective the SDGs have been in encouraging organizations to become more sustainable. Addressing this gap begins with understanding the antecedents that motivate organizations to pursue SDGs. Few studies have considered such antecedents and those that did have focused on a limited set of goals (Magliacani, 2022; Mio et al., 2020; Sullivan et al., 2018). The scarcity of studies examining organizations’ engagement with the complete set of SDGs calls for a broader analysis of whether and to what extent SDGs have successfully raised firms’ awareness of global sustainability challenges.

To answer this call, the present study focused on the extent to which companies engage in SDGs, which was measured in four different ways: the total number of SDGs, the total number of environmental SDGs, the total number of social SDGs, and the total number of a focused subset of SDGs (i.e., SDG8, SDG12, and SDG13). Through a content analysis of the reports from the top 100 most sustainable companies in the world, we observe that firms’ attention toward SDGs has particularly gravitated around SDG8 (Decent work and economic growth), SDG12 (Responsible consumption and production) and SDG13 (Climate action). On the contrary, little attention has been paid to aspects such as defeating poverty (SDG1), ending hunger (SDG2), and conserving the oceans, seas, and marine resources (SDG14). These findings are consistent with van Zanten and van Tulder’s (2018) argument that companies are more likely to engage in goals that they see as actionable (i.e., they have more competencies and tools to address the goals). Our study contributes to the literature on SDGs by refining their argument and empirically demonstrating that SDG8, SDG12, and SDG13 are the most broadly actionable goals.

Given that not all SDGs have equally succeeded in raising companies’ attention to sustainability challenges, we need to deepen our understanding of the antecedents of SDG engagement. To this end, the present study drew on neo-institutional theory to explore how institutional pressures have affected organizations’ SDG engagement. Past studies have emphasized that institutional pressures play a central role in encouraging corporations to adopt responsible behaviors toward the economy, society, and the environment (Bansal, 2005; Marano & Kostova, 2016; Rasche et al., 2013; Roman, 2017). Similarly, we suggest that two sources of institutional pressure affect the breadth of SDGs that firms pursue.

The first source is country-of-origin: in particular, developing vs. developed countries. The extant literature highlights the dissimilar institutional pressures in these countries, with the former suffering weaker governmental and social pressures relative to the latter (Cordeiro et al., 2018, 2021; Earnhart et al., 2014). Thus, we expected that weaker institutional pressures would provide little incentive for organizations to invest in pursuing SDGs, which would be reflected in their commitment to a narrower set of SDGs. Contrary to our hypothesis, we found that institutional pressures from developing countries are positively associated with SDG engagement. Specifically, pressures on companies in developing countries explain both a general communication strategy and a focused communication strategy. These findings suggest that firms headquartered in developing countries are more proactive about reporting their investments within the SDG framework. In other words, we observe that the domestic regulations and cultural contexts of developing countries exert more coercive pressures on their firms than those in developed countries. Our approach contributes to the SDG literature by addressing Mio et al. (2020) and Bashir and Qureshi (2022) call for more studies that distinguish between developing vs. developed countries. In doing so, we provide novel empirical evidence that developing countries have been more receptive to SDGs’ call to action than their developed peers. This may be due to the new regulatory actions recently undertaken by local governments in developing countries to encourage firms to participate in sustainability-related initiatives. For example, the South Korean domestic government presents sustainability-related initiatives as a form of quasi-tax (Global Compact Network Korea, 2010), whereas the Indian government (via the 2013 Companies Act) has mandated that large firms invest at least 2% of their sales in sustainability initiatives (Cordeiro et al., 2018).

The industry type is the second source of institutional pressure that may affect the breadth of SDGs that firms pursue. In recent years, there has been exponential growth in the demand for firms to reduce the impact of their activities on the environment (Gunawan et al., 2020; Li et al., 2018; Liute & de Giacomo, 2022). With both governments and societies becoming less tolerant of high-polluting firms, we expected that industries that record higher levels of pollution would disclose a broader range of SDGs to improve their legitimacy. By combining data on industry type with the aforementioned content analysis, we affirmed that institutional pressures from high-polluting industries are positively associated with the breadth of SDGs. These findings are consistent with Alonso-Martínez et al.’s (2020) evidence that corporate social performance is positively related to pollution. Therefore, our results further expand the literature on SDGs by demonstrating that companies operating in high-polluting industries commit to a broader range of SDGs in an attempt to achieve legitimization.

Finally, we evaluated whether and how companies’ engagement in the whole set of SDGs or a specific subset (i.e., SDG8, SDG12, and SDG13) affects their financial performance. We found that SDG engagement is positively related to companies’ financial performance, regardless of the focus on the whole set of SDGs or a specific subset. Our evidence indicates that stakeholders reward either a general engagement or a focused engagement on a small set of SDGs. These results contribute to the discussion on the symbolic vs. substantive nature of SDGs (Elalfy et al., 2020; Silva, 2021).

Our research also has implications for managers and policymakers. First, our findings highlight the potential challenges of adopting SDGs. Some SDGs are less frequently addressed in general, while attention on other SDGs wavers based on factors such as industrial pollution level and country status (i.e., developed vs. developing). These results suggest that SDGs have generally enhanced firms’ awareness about the importance of adopting responsible behaviors, but much more work is needed to mobilize their efforts at addressing a broader array of issues. Specifically, we found that SDG1 (No poverty), SDG2 (Zero hunger), SDG14 (Life on land), and SDG15 (Life underwater) are the least addressed goals in general and in each category. There are several possible explanations for these results. One is that those goals are technically achieved through other goals and thus less communicated in CSR reports. Another is that the goals are too complex for companies to address themselves, thereby leading companies to entrust the issue to other institutions. If this latter explanation is accurate, policymakers should develop initiatives to support companies in addressing these goals.

Second, by pursuing CSR reporting within the SDG framework, companies signal that they are responsible corporate citizens to stakeholders. Thus, managers should see SDGs as an important opportunity to bolster their firms’ financial performance. To this end, managers can adopt two possible communication strategies. First, they can disclose information on a broad swath of SDGs to demonstrate their consideration of all interests and stakeholders. Alternatively, they can emphasize the most frequently addressed SDGs (SDG8—Decent work and economic growth; SDG12—Responsible consumption and production; SDG13—Climate action), for which they likely have the most power and competency to address.

### Limitations and future research

Our research has several limitations that represent opportunities for future research. In this study, we mostly understood the role of institutional pressure through regulations and cultural groups. However, as the sample encompassed global companies that are well-known in the institutional context, it is important to also consider how these companies have shaped the institutions that influence their behaviors (Donaldson & Dunfee, 1999; Verbeke et al., 2018). Moreover, as the 17 SDGs incorporate 169 targets that are linked to each other and sometimes even overlap, our focus on goals rather than targets may obscure important information about why some goals are pursued more than others. Another limitation of this study concerns our measure of SDG disclosure. In our categorization of SDGs as either environmental or social initiatives, we excluded SDG9 (Industry, innovation, and infrastructure) and SDG17 (Partnerships for the goals) because of the challenges associated with placing them in the most adequate categorization. Future research may apply different criteria to categorize SDGs to further investigate the antecedents and financial outcomes of SDGs. Finally, scholars should incorporate a broader array of institutional pressures and financial performance metrics to deliver deeper insights into how and why companies engage with SDGs.
